# The mechanisms of immune response and evasion by the main SARS-CoV-2 variants

**DOI:** 10.1016/j.isci.2022.105044

**Published:** 2022-09-02

**Authors:** Qiuli Chen, Jiawei Zhang, Peter Wang, Zuyong Zhang

**Affiliations:** 1 The Affiliated Hangzhou Xixi Hospital, Zhejiang Chinese Medical University, Hangzhou, Zhejiang 310023, China; 2 Zhejiang Chinese Medical University, Hangzhou, Zhejiang, China; 3 Department of Research and Development, Zhejiang Zhongwei Medical Research Center, Hangzhou, Zhejiang 310018, China

**Keywords:** Immunology, public health, virology

## Abstract

Coronavirus disease 2019 (COVID-19) caused by severe acute respiratory syndrome coronavirus 2 (SARS-CoV-2) has caused a global pandemic. SARS-CoV-2 carries a unique group of mutations, and the transmission of the virus has led to the emergence of other mutants such as Alpha (B.1.1.7), Beta (B.1.351), Gamma (P.1), Kappa (B.1.617.1), Delta (B.1.617.2) and Omicron (B.1.1.529). The advent of a vaccine has raised hopes of ending the pandemic. However, the mutation variants of SARS-CoV-2 have raised concerns about the effectiveness of vaccines because the data showed that the vaccine was less effective against mutation variants compared to the previous variants. Mutation variants could easily mutate the N-segment structure and receptor domain of its spike glycoprotein (S) protein to escape antibody recognition. Therefore, it is vital to understand the potential immune response and evasion mechanism of SARS-CoV-2 variants. In this review, immune response and evasion mechanisms of several SARS-CoV-2 variants are described, which could provide some helpful advice for future vaccines.

## Graphical abstract

**Figure:**
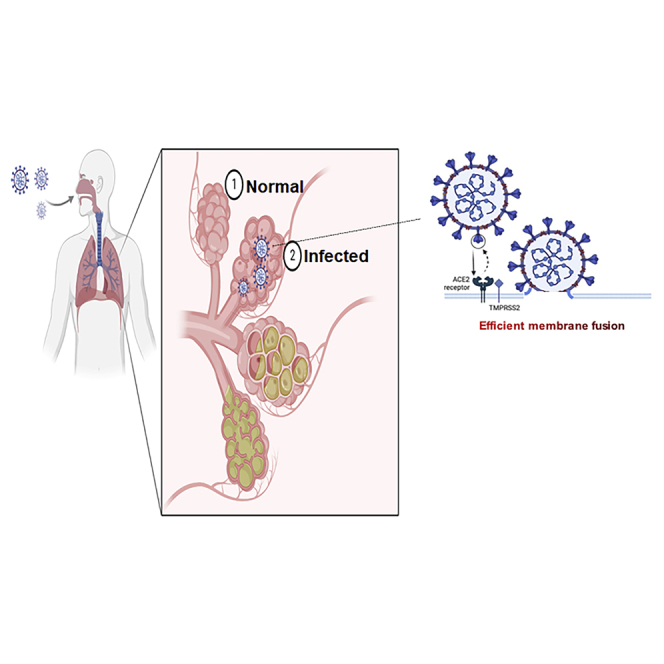

## Background

In December 2019, the severe acute respiratory syndrome coronavirus 2 (SARS-CoV-2) was first discovered in Wuhan, Hubei, China. Later, the World Health Organization (WHO) declared that coronavirus disease 2019 (COVID-19) caused by the SARS-CoV-2 led to a global pandemic (Dong et al., 2020). The spread of COVID-19 has brought catastrophic losses, such as hitting economies and taking away millions of lives. According to WHO (https://covid19.who.int/), as of June 8, 2022, there have been 536,575,203 confirmed cases and 6,323,378 deaths globally. Worse still, the SARS-CoV-2 carries a unique group of mutations and the transmission of the virus has led to the emergence of other mutants such as Alpha (B.1.1.7), Beta (B.1.351), Gamma (P.1), Kappa (B.1.617.1) and Delta (B.1.617.2) (Table 1) (Dong et al., 2021; Dyer, 2021). These variants differ from each other in several characteristics, such as transmissible ability, ease of penetration of somatic cells, and resistance to immune system responses (Table 2) (Liu et al., 2021).

**Table 1:** Lineages of SARS-CoV-2 variants

Lineage	WHO label	First detected (when)	First detected (where)
B.1.1.7	Alpha	18 December 2020	United Kingdom
B.1.351	Beta	May 2020	South Africa
P.1	Gamma	November 2020	Brazil
B.1.617.2	Delta	October 2020	India
B.1.1.529	Omicron	November 2021	Multiple countries

**Table 2:** Molecular mechanisms of immune evasion by the SARS-CoV-2 variants

Lineage	Mutations	Functions	Mechanisms	Ref
B.1.1.7	N501Y E484K	Mutation 501 Y enhances the affinity of RBD to ACE2; A π-π interaction enhances RBD binding to ACE2 and eliminates potent neutralizing antibody binding.	Perform immune escape by disrupting antibody binding; the upregulating of Orf6 and Orf9b has contributed to the increased ability of immune evasion.	(Starr et al., 2020; Supasa et al., 2021; Yang et al., 2021; Thorne et al., 2022)
B.1.351	K417N E484K N501Y	N501Y and E484K have increased binding of RBD and ACE2.	N501Y and E484K have increased binding of RBD and ACE2.	(Zhou et al., 2021)
B.1.1.28	N501Y E484K	Resistance to neutralization by nAb, including naturally acquired and vaccination-induced resistance.	E484K and N501Y increase the binding affinity of S protein to ACE2.	(Gobeil et al., 2021; Tegally et al., 2020)
B.1.617.2	L452R	S2X303 can cross-react with multiple mutant strains compared with other neutralizing antibodies.	The L452R has a stronger affinity for ACE2 receptor and reduces the recognition ability of the immune system.	(McCallum et al., 2021c; Zhang et al., 2021)
B.1.1.529	K417N, G446S, E484A, Q493R	Mutations can cause immune evasion of the novel coronavirus. Omicron strains have enhanced spread owing to enhanced S protein consistency.	Omicron Spike Trimer has enhanced stability. K417N, G446S, E484A, and Q493R bypass neutralizing antibodies whose epitopes overlap with the ACE2-binding motif.	(Fang and Shi, 2022; Johnson et al., 2021; Shuai et al., 2022; Cui et al., 2022)

Based on the WHO, the variant strains of COVID-19 are classified into two categories based on their risk: VOC (Variant of Concern) and VOI (Variant of interest). The former causes a large number of cases worldwide, with data confirming its transmissibility and virulence, reducing the effectiveness of vaccines and clinical treatments. The latter has been confirmed in cases of community transmission and detected in multiple countries, but has not yet become widespread. At present, VOC is the most influential and global threat to the epidemic variation strains, including Alpha, Beta, Gamma, Delta, and Omicron. The Alpha strain spread rapidly in late 2020 and became the dominant mutant strain in the first half of 2021. After Alpha, Beta is very strong in immune escape, leading to high infectious ability. The Gamma variant was first detected in Brazil in November 2020 and accounted for 76% of COVID-19 cases in South America in June 2021, which is the most dominant strain in South America.

In 2021, Delta mutant virus has become the dominant virus strain in the global COVID-19 pandemic owing to its higher transmissibility, which initially appeared in India in October 2020 and caused a new wave of infections globally (Wheatley and Juno, 2022). Instead of staying in India, the rapidly spreading Delta variant has invaded more than 92 countries from far East Asia to America, Europe, and Africa. As of 23 November 2021, Delta variant has led to 950,000 confirmed cases in the United States, 820,000 cases in the United Kingdom, and 110,000 cases in Germany (Haldane et al., 2021). Omicron has caused a wave of diseases and deaths in places all over the world as it was first discovered in late 2021 in South Africa. It is believed that the risk of omicron virus infection was 48% higher than the risk of Delta virus infection because Omicron proliferates are about 70 times faster in the bronchi than in the Delta variant (Mallapaty, 2022). The extremely high rate of transmission, combined with its ability to evade double vaccination and the body’s immune system, means that the total number of infections remains worrying (Gowrisankar et al., 2022).

Omicron has developed distinctive mutations as a result of rapid expansion and numerous possibilities (Hui et al., 2022). Therefore, several sublineages or sub-variants have been reported. The first two sub-variants were given the designations BA.1 and BA.2. BA.1.1, BA.2.12.1, BA.2.3, BA.2.9, BA.3, BA.4, and BA.5 have now been added to the existing list and some variants have become the globally dominant variants (Yamasoba et al., 2022; Xia et al., 2022). One study discovered that BA.1 and BA.2 share 32 identical mutations, whereas BA.1 and BA.2 have 28 different mutations (Yu et al., 2022). An epidemiological study has revealed that during the BA.2 epidemic, the infection rate in the UK was 6.37%, which was the highest infection rate in previous outbreaks. The BA.2 sublineages include BA.2.12.1 and BA.2.13. In the BA.2 epidemic, the elderly have the most obvious increase in infection, so the BA.2 epidemic still leads to a high mortality rate (Elliott et al., 2022). The competitive advantage of the Omicron mutant strain is significantly higher than that of the previous strain Delta, reaching the high infectivity region of all viruses in history. BA.3 has exhibited limited transmissibility, but BA.4 and BA.5 have caused many infection cases in some countries (Xia et al., 2022). The Omicron strain is currently called the mainstream strain, and may still be the key strain in the later stage (Elliott et al., 2022).

This article focused on the immune escape mechanisms of the two dominant variants: Delta and Omicron, because they are the most rapidly and widely circulating dominant strains recently. Meanwhile, this article has summarized the immune escape mechanism of other high-concern strains to comprehensively provide lessons for better response to the epidemic in the future.

## Structural biology

SARS-CoV-2, an enveloped RNA virus, targets the host cell membrane via fusion of lipid bilayer by the trimeric spike glycoprotein (S) after S protein interacts with angiotensin-converting enzyme 2 (ACE2) on the host cell surface, leading to entering a host cell (Shang et al., 2020). The S protein is a single-chain homotrimer, which consists of the receptor-binding S1 and the fusion S2 subunit (Hu et al., 2021). The protein has two functional domains: one is the S1 receptor binding domain (RBD) and the other is the S2 domain that mediates the fusion of viral and host cell membranes (Walls et al., 2020). The S protein can be cleaved by a furin-like protease when it binds to ACE2, leading to S1 dissociation and S2 conformational change and subsequent membrane fusion between the virus envelope and host cell membrane (Wu et al., 2020). Cleavage of Furin at S1/S2 leads to conformational changes in the viral S protein that expose the RBD and/or S2 domains. TMPRSS2 cleavage of SARS-CoV-2 S promotes fusion of the viral capsid with the host cell, allowing entry of the virus (Bestle et al., 2020; Yuan et al., 2020). It has been known that S1 comprises four domains, designated N-terminal domain (NTD, residues 14-305), receptor-binding domain (RBD, residues 328-531), and two C-terminal domains (CTDs) (Peng et al., 2021). Functionally, NTD binds the attachment factors, such as AXL, L-SIGN, and DC-SIGN, and RBD interacts with ACE2, and CTDs protect the pre-fusion S2 from the host cell immune system (Kielian, 2020). The S2 subunit contains heptad repeat HR1 and HR2 and the fusion peptide, which make S2 modify their conformation to initiate fusion and enter host cells and cause infection (Shang et al., 2020). In addition, cathepsin 1 also mediated the cleavage of the S2 site and allow viral entry of SARS-CoV-2 (Hoffmann et al., 2020).

## Various SARS-CoV-2 variants

The sequence of the original SARS-COV-2 strain was collected in Wuhan on December 24, 2019 (Kumar et al., 2021a). To estimate the coronavirus type of human progenitors, Chinese researchers compared these early genomes to bat and pangolin coronavirus strains; the ancestral genomic type was recognized as "S" and the dominant derivative type was designated as "L" to reflect the mutant amino acid alterations (Tang et al., 2020). Similar investigations were carried out by some scholars, the ancestral type was named "A" and the developed type was labeled "B" which is the ancestor of the main global concern variety (Forster et al., 2020; Rambaut et al., 2020a).

## Epsilon/B.1.427/B.1.429

In California, the SARS-CoV-2 B.1.427/B.1.429 variant was first identified, and this variant has spread to more than 34 countries as of June 2021 (Deng et al., 2021; Rambaut et al., 2020a). It was been demonstrated that the S mutations of both B.1.427 and B.1.429 are the same, including signal peptides S13I, NTD W152C, and RBD L452R (Deng et al., 2021). The ancestors of two mutations emerged in May 2020 and gradually diverged into B.1.427 and B.1.429 in June and July 2020. Cases related to these mutations rose rapidly and they were considered a type variant of concern by CDC (Control and Prevention, 2021). Plasma neutralization activity was determined by collecting plasma from 15 individuals who received two doses of the Moderna mRNA-1273 vaccine and 15 individuals who received two doses of the Pfizer/BioNtech BNT162b2 vaccine. Notably, the neutralizing activities of vaccine-elicited and infection-elicited Abs were reduced by three mutations present in the B.1.427/B.1.429 S glycoprotein, suggesting that residues of this lineage are involved in immune evasion (McCallum et al., 2021a).

**Figure 1:**
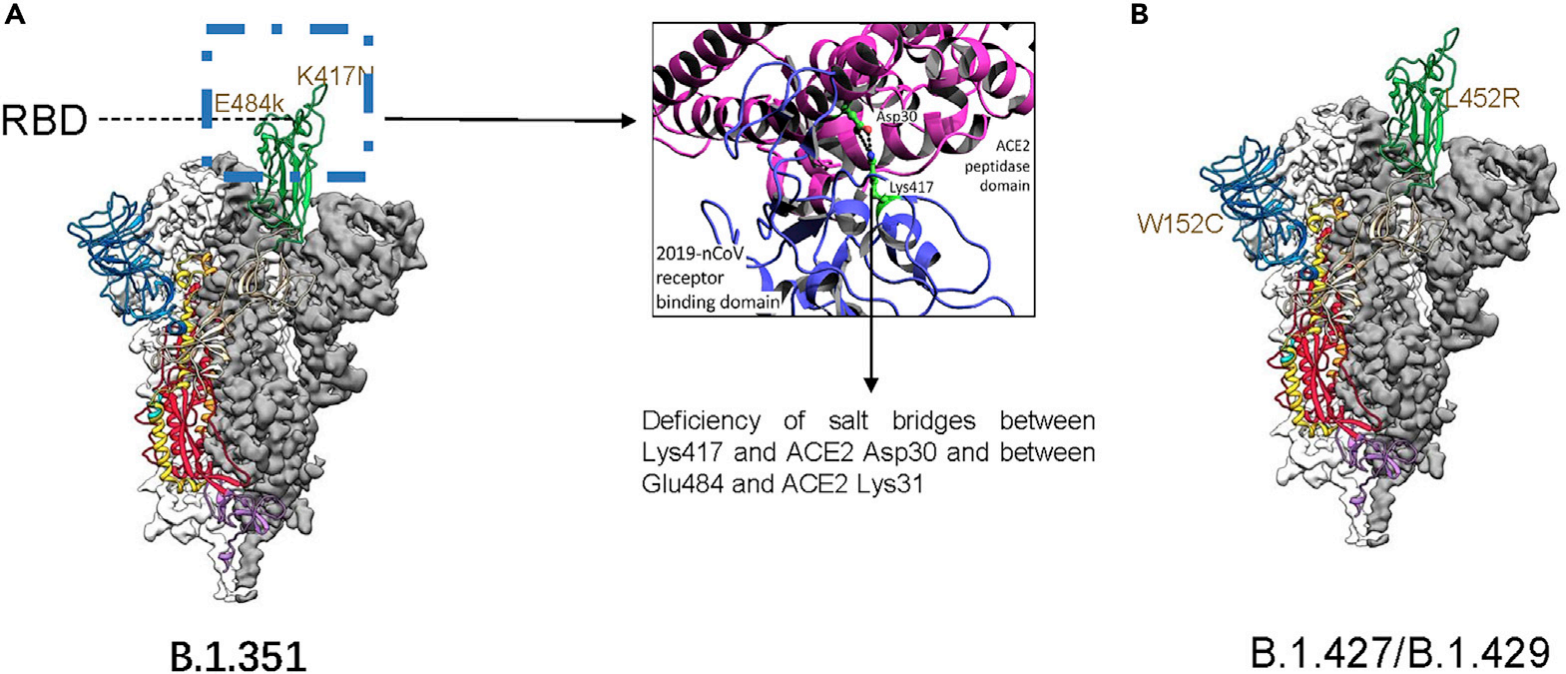
Mutations of B.1.1.7Alpha, Epsilon/B.1.427/B.1.429 (A) lose binding and neutralization owing to E484K and K417N; deficiency of salt bridges between Lys417 and ACE2 Asp30 and between Glu484 and ACE2 Lys31. (B) S mutations of B.1.427 and B.1.429, including signal peptides S13I, NTD W152C, and RBD L452R.

Moreover, it is noteworthy that the sensitivity of RBD- and NTD-specific Abs was reduced because of the S mutation in B.1.427/B.1.429. More specifically, Biolayer interferometry results showed that 10 RBD-specific mAbs with a 10-fold or more reduction in neutralization potency of the B.1.427/B.1.429 variant relative to D614S bound poorly to the L452R RBD mutants (McCallum et al., 2021a). The neutralizing activity of all 10 NTD-specific mAbs was abolished owing to the presence of S13I and W152C mutations (Figure 1). These data suggest that the reduced neutralization potency of the B.1.427/B.1.429 variant is owing to evasion of neutralization mediated by RBD- and NTD-specific mAbs (McCallum et al., 2021a). Biolayer interferometry results proved that the B.1.427/B.1.429 variant had reduced neutralization potency because the D614S has a poor binding with L452R RBD mutations, which indicated a direct role of this mutation in immune evasion (McCallum et al., 2021a).

## B.1.1.7Alpha

The B.1.1.7 lineage was discovered in the UK in September 2020, and the WHO has marked it as Alpha and defined it as a variant of concern (Washington et al., 2021; Galloway et al., 2021). The Alpha variant has 23 mutations, including 17 protein sequence changes, and 6 synonymous mutations (Rambaut et al., 2020b). The spike protein plays a crucial role in cell-cell contact transmission, and genetic mutations in the S protein may alter clinical features, such as viral infectivity, pathogenicity, and antibody response. There are 8 additional mutations within the S protein in the Alpha variant, including the most concerned mutations—the asparagine (N) to tyrosine (Y) mutation of 501 position, which enhances the affinity of RBD to ACE2 (Yang et al., 2021; Starr et al., 2020). Despite the increased binding of ACE2, the mutations will also cause viral immune evasion and this is confirmed by another study (Yang et al., 2021). By cryo-electron microscopy (cryo-EM), it can be observed that the receptor binding domain of the B.1.1.7 variant is open status. The A570D mutation can regulate RBD on and off. The N501Y mutation introduces a π-π interaction that enhances RBD binding to ACE2 and eliminates potent neutralizing antibody (nAb) binding (Yang et al., 2021). Furthermore, other accumulated evidence from genomic and proteomic studies also confirmed that the B.1.17 variant binds ACE2 receptors with 5-fold and 2-fold higher affinity than D614G S RBP and wild-type S RBD, respectively (Gobeil et al., 2021; Ramanathan et al., 2021). At the same time, another study suggests that the N501Y mutation in the B.1.1.7 variant can perform immune escape by disrupting antibody binding. This phenomenon is particularly evident in mAb 278 with a 78% neutralization rate (Supasa et al., 2021).

S982A and A570D transformations of the B.1.1.7 variants have concentrated on an outward shift of the CTD1, accordingly loosening up the fusion peptide proximal region (FPPR) and the 630 loop, which assist with holding the RBD in its "down" position in the parental strain (Chemaitelly et al., 2021). The transformations increment the recurrence with which the S trimer tests the RBD-up compliance, permitting B.1.1.7 to more readily introduce the receptor restricting theme (RBM) to ACE2 on the host cells (Chemaitelly et al., 2021). When one RBD flips up, the completely or somewhat requested 630 loops of the adjoining protomers settle the CTD2, which the overlap along with the N-terminal section of S2, and consequently forestalls the untimely S1 separation. N501Y additionally expands the liking of that area for the receptor, and it is most likely owing to the hydrophobic connection of Tyr501 with Tyr41 of ACE2 and potential cation-π communication with ACE2 Lys353 (Chemaitelly et al., 2021).

The blend of improved RBM and extra nearby communications may permit the B.1.1.7 infection to contaminate cell types with lower ACE2 levels than those of the nasal and bronchial epithelial cells that the infection normally taints; an extended cell tropism could represent the expanded danger of mortality in patients tainted with this variation. Cells with lower ACE2 levels were more susceptible to infection with B.1.1.7 viruses, possibly owing to a combination of enhanced RBM performance and additional local interactions. The expanded danger of death in transformed patients could be clarified by broadened cell tropism (Zhang et al., 2021; Challen et al., 2021). There are no major adjustments in the RBD and NTD caused by the transformations in B.1.1.7. Consistently, the change is similar to the sensitivity of potently neutralizing antibodies (Abdool Karim and de Oliveira, 2021). In terms of B.1.351, the structure of the G614 trimer was largely maintained in S proteins and they own similar biochemical constancy. There are no large changes caused by E484K, N501Y, and K417N, and an increase in receptor affinity conferred by N501Y is attenuated by the deficiency of salt bridges between Lys417 and ACE2 Asp30 and between Glu484 and ACE2 Lys31 (Figure 1). It is believed that antibodies against the RBD-2 epitope will lose binding and neutralization owing to E484K and K417N. The B.1.351 variant changed the two major neutralization sites on the S trimer, and some studies suggest that the B.1.351 variant might have been selected because the ability to make contact with host cells is only slightly compromised (Cai et al., 2021).

**Figure 2:**
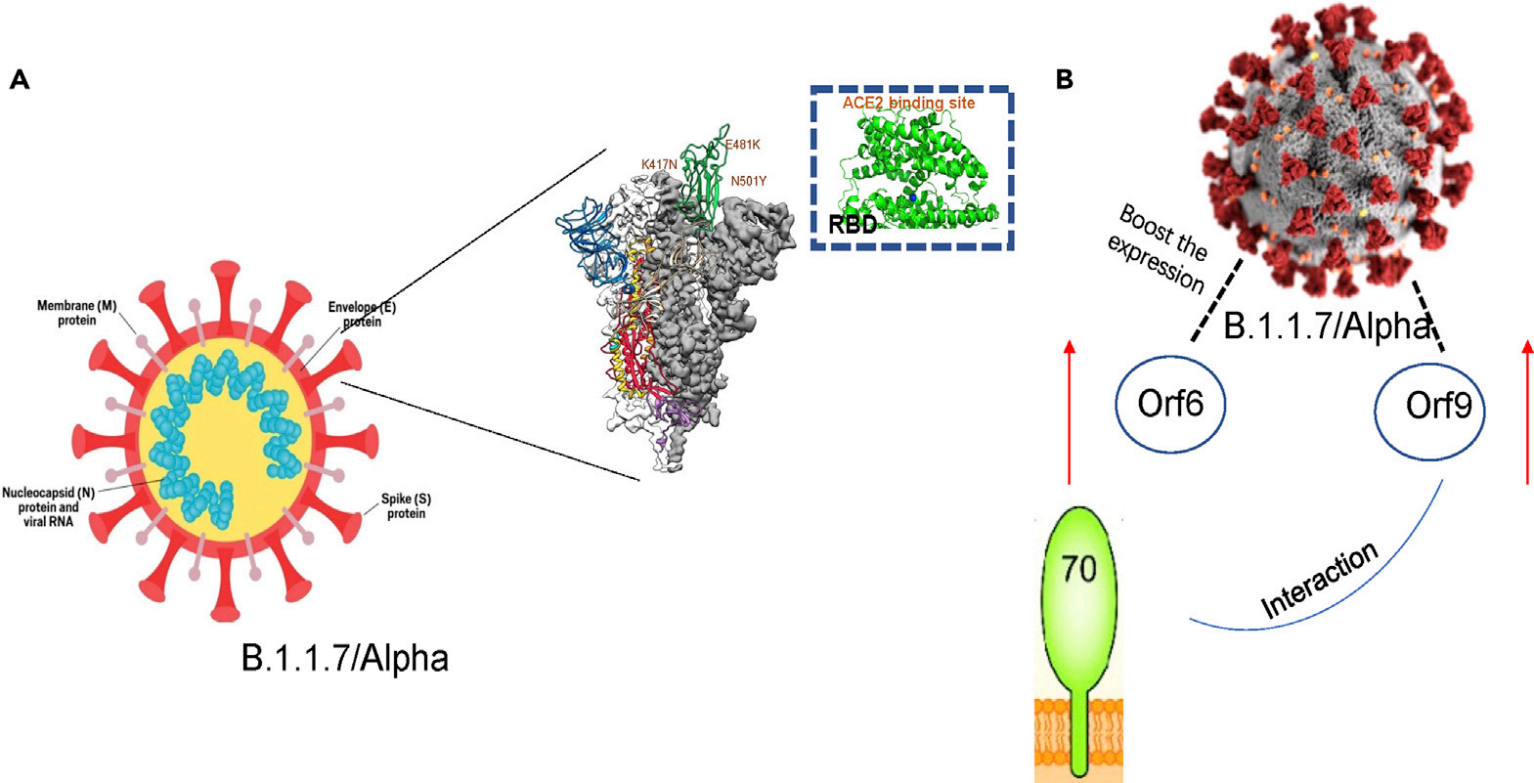
Immune escape mechanism of B.1.1.7Alpha (A) N501Y mutation in the B.1.1.7 variant can perform immune escape by disrupting antibody binding. (B) B.1.1.7 boosted the expression of Orf6 and Orf9 and the innate immune response is suppressed by Orf9b expression alone and its interaction with TOM70. Upregulation of Orf6, and Orf9b has contributed to an increased ability of immune evasion.

In the B.1.1.7 lineage, it was found that this strain significantly boosted the expression of Orf6 and Orf9b. It is believed that Orf6 and Orf9b belong to innate immune antagonists and it is worth noting that Orf6 and Orf9b are associated with the ability of immune evasion in the B.1.1.7 lineage (Thorne et al., 2022). Notably, the authors hold the opinion that the inhibition of Orf9b on innate immunity may be mediated by the host’s innate response itself. More specifically, the innate immune response is suppressed by Orf9b expression alone and its interaction with TOM70 (mitochondrial preprotein responsible for recognition and transport of cytoplasmic synthesis). The upregulation of Orf6 and Orf9b nucleocapsid proteins has contributed to an increased ability of immune evasion. More specifically, The Alpha version was found to have significantly higher levels of subgenomic RNA and protein levels of Orf9b, and Orf6. Through interaction with TOM70, Orf9b expression alone reduced the innate immune response (Lin et al., 2010). Furthermore, phosphorylation controlled Orf9b activity and its connection with TOM70 (Thorne et al., 2022). Moreover, this study suggested that Alpha-enhanced innate immune antagonism is beneficial to the ability of dissemination, which will help boost replication through the way to diminish or delay early the innate responses of host cells (Figure 2) (Thorne et al., 2022).

## B.1.351/beta

The B.1.351 lineage, also named Beta variant by the WHO, was first discovered in South Africa in May 2020 and quickly became the dominant strain in South Africa (Baral et al., 2021). The Beta variant has a total of nine mutations: L18F, D80A, D215G, R246I, K417N, E484K, N501Y, D614G, and A701V (Wibmer et al., 2021; Mwenda et al., 2021; Wu et al., 2021). RBD mutations in the Beta variant include K417N, E484K, and N501Y, which may lead to immune evasion of mAb (Baral et al., 2021; Zhou et al., 2021). Neutralizing capacity for B.1.351 remains in many convalescent and vaccine sera. Many convalescent and vaccine sera still have some neutralizing ability to B.1.351. At the same time, some monoclonal antibodies can effectively neutralize B.1.351 (Zhou et al., 2021).

**Figure 3:**
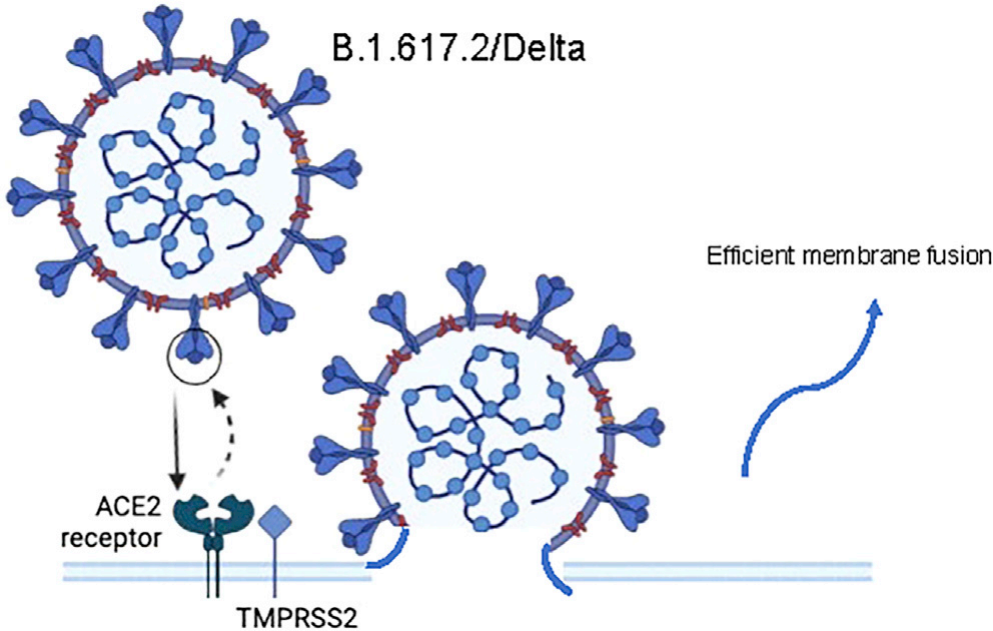
Immune escape mechanism of B.1.617.2/Delta Efficient membrane fusion ability led to immune evasion.

Wang et al. found that the Beta variant did not show higher infectivity in hACE2-type cells but showed higher infectivity in mACE2-type cells (Li et al., 2021). The Beta variants are able to escape the most neutralizing mAbs recognition. More specifically, in the Beta variant, increasing mutation sites of RBD are associated with its immune evasion from a rising quantity of monoclonal antibodies. In order to further study the mechanism of immune evasion, 17 monoclonal antibodies against the Beta strain were divided into 5 groups according to the mutation sites. Concisely, K417N mutation can lead to the escape from 157, 2H10, and 1F9 antibodies; E484K mutation can lead to the escape from 261 to 262, 9G11, P2B-2F6, and LKLH; N501Y mutation can lead to escape from H00S022 and 10F9; K417N and N501Y can lead to the escape from 10D12, 11D12, 247 (Figure 3) (Li et al., 2021).

## B.1.1.28/gamma (P.1)

The P.1 lineage was also named Gamma by the WHO. It was first detected in Brazil in November 2020 and was defined as a variant of concern by the WHO on 11 January 2021 (Salleh et al., 2021). The Gamma variant spread to the United States, Canada, and Belgium, but its transmission capacity is unknown. Gamma variants have multiple mutations in S protein: L18F, T20N, P26S, D138Y, R190S, H655Y, T1027I, V1176F, K417T, E484K, and N501Y (Faria et al., 2021). It is worth mentioning that the gamma variant has several key mutations: K417T, E484K, and N501Y (Tegally et al., 2020; Gobeil et al., 2021). The other mutations that define the P.1 variant are five substitution mutations in NTD (L18F, T20N, P26S, D138Y, and R190S) and three substitutions in the S2 subunit (D655Y, T1027I, and V1176F) (Wise, 2021). For Beta and Gamma, the interaction pattern of ACE2 is basically the same. Meanwhile, NTD of the Gamma variant has five mutations: R190S, L18F, T20N, P26S, and D138Y and other three subunit mutations are found, including D655Y, T1027I, and V1176F (Salleh et al., 2021). However, it has been suggested that the Gamma variant has more mutations than Beta, but its resistance to neutralization by nAb, both naturally acquired and vaccination-induced resistance, is effectively reduced. Nevertheless, the RBD-target mAb may express a decreased or complete loss of neutralization of Gamma variants such as LY-CoV555 nAb (Salleh et al., 2021).

## B.1.617.1 (Kappa) and B.1.617.2/Delta

The B.1.617.2/Delta variant is considered the most infectious of all variants (Baral et al., 2021). It was first detected in India in October 2020 and has since become the dominant strain globally (Vaughan, 2021). The ability of the viral spike protein to bind and enter host cells can be altered by mutations in the RBD. The S protein mutations include: T19R, G142D, Δ156-157, R158G, L452R, T478K, D614G, P681R, and D950 N (Control and Prevention, 2021). A study from Dr. David Veesler’s group was published in Science to demonstrate how the Delta strain of coronavirus avoided being recognized by host antibodies (McCallum et al., 2021c). The article proposed that mutations in the S protein of B.1.617.1 (Kappa) and B.1.617.2 (Delta) disrupt the recognition of some monoclonal antibodies by altering key antigenic sites, including NTD remodeling in Delta strains (McCallum et al., 2021c). The affinity of Kappa and Delta RBD to the ACE2 receptor was similar to that of the original variant, while the affinity of B.1.617.2+ (Delta+) was significantly reduced. The study collected plasma samples from 37 participants who received either two doses of Moderna mRNA-1273 or Pfizer/BioNtech BNT162b2 or one dose of Janssen vaccine Ad26.COV2.S (McCallum et al., 2021c). The research results indicated that the Delta, Kappa, and Delta + mutants were able to neutralize the viral neutralization potential of the antibodies induced by the virus, and the Delta + mutants caused the greatest reduction in the efficacy of the antibodies (McCallum et al., 2021c).

Besides, from laboratory tests, half of the Janssen vaccine recipients completely lost the ability to neutralize one or more mutant strains. At the same time, compared with other strains, Delta and Kappa mutant strains can better avoid the neutralizing potential of antibodies induced by vaccines (McCallum et al., 2021c). Then, the researchers used cryo-EM to observe that the Delta mutant strain produced a special molecular strategy to reduce antibody sensitivity. In addition, the researchers have discovered a particular antibody, S2X303, which can cross-react with multiple mutant strains compared with other neutralizing antibodies. Moreover, this new antibody can form a special contact footprint in the NTD antigenic supersite (McCallum et al., 2021c).

Importantly, Bing Chen and his team analyzed the structure of the S protein of the mutated virus and elucidated the differences in membrane fusion of different mutated strains, providing more details for understanding the dynamics of virus infection (Zhang et al., 2021). In order to explore whether the high infectivity of the Delta mutant is owing to the more efficient membrane fusion of the S protein, the intercellular fusion test was conducted. The results showed that the fusion efficiency of the Delta S protein was significantly higher than that of other strains after a long period of time and even in infected HEK293 cells with low ACE2 expression (Zhang et al., 2021). Moreover, pseudovirus neutralization test also showed that the Delta variant strain could establish infection in 60 min, which was significantly faster than other strains. SDS-PAGE was used to analyze the changes in three conformations (pre-fusion S-trimer, post-fusion S2 trimer, and dissociated S1 monomer) of the S proteins of different mutant strains. It was found that Delta S proteins only had a single pre-fusion trimer conformation, unlike other mutant strains. The binding ability of S protein trimer to ACE2 and anti-S protein antibody was quantitatively determined by biofilm layer interference technology (Zhang et al., 2021). The binding of the Gamma mutant was significantly stronger than that of the original strain, which was mainly owing to the mutations of K417T, E484K, and N501Y in its RBD region, and the affinity of the Kappa and Delta mutants was between the original strain and the Gamma strain (Zhang et al., 2021). However, the Delta variant did not show high ACE2 binding ability, which may be caused by ACE2-induced dissociation of S1. The ability of the mutants to bind antibodies was changed to some extent. In the Delta mutant, the binding ability of two antibodies targeting the spike protein NTD-1 was lost, and the neutralization activity was similarly changed. Overall, the Kappa and Gamma mutants had a stronger immune escape ability against antibodies than the Delta strains (Zhang et al., 2021).

The full-length spike protein trimer was analyzed using cryo-EM by another group (Zhang et al., 2021). Asn343 glycosylation was evident in all mutants, and the polysaccharide was able to bind with adjacent RBD and stabilize the 3-RBD-downward conformation. Meanwhile, the FPPR plays an important role in the regulation of S protein stability and structural rearrangement, and conformational changes occur in this region in the Delta mutant (Zhang et al., 2021). Compared with the original strain, the most significant difference in the Delta mutant was in the NTD portion of the S protein, which contained three point mutations (T19R, G142D, and E156G) and two deletion mutations (F157del and R158del). The mutations resulted in significant changes in the epitopes near NTD-1. Mutations of L452R and T478K in the RBD region did not have a significant effect (Zhang et al., 2021). Based on the above findings, the researchers believe that the main reason for the dominance of Delta virus is its efficient membrane fusion ability rather than its high affinity for receptors (Figure 3).

## B.1.1.529/Omicron

The sequence of the Omicron variant of novel coronavirus was first elucidated in November 2021 (He et al., 2021). One study found that Omicron has more than 30 mutations in the S protein, including 15 mutations in the receptor binding domain (RBD) (Flemming, 2022). Mutations include: T91, P13L, E31del, R32del, S33del, R203K, G204R, D3G, Q19E, A63T, N211del/L212I, Y145del, Y144del, Y143del, G142D, T95I, V70del, H69del, A67V, Y505H, N501Y, Q498R, G496S, Q493R, E484A, T478K, S477N, G446S, N440K, K417N, S375F, S373P, S371L, G339D, D796Y, L981F, N969K and Q954H (Faria et al., 2021). One study indicated that many of these RBD mutations are thought to reduce the potency of neutralizing antibodies (Grabowski et al., 2022; Callaway and Ledford, 2021). G339D, S373P, S375F, K417N, N440K, S477N, T478K, E484A, Q493R, Q498R, N501Y, and Y505H are among the 12 mutations shared by BA.1 and BA.2. S371L, G446S, and G496S were only found in BA.1, although R346K was discovered in one of these members, BA.1.1. BA.2 has two distinct RBD mutations, S371F and R408S, and shares T376A and D405N with BA.3 (Fan et al., 2022). BA.2.12.1 and BA.4/BA.5 were further improved by 25 and 10% compared to the current popular Omicron BA.2. The significant increase in immune escape may be owing to the introduction of the L452 mutation. The BA.2 sublineages, including BA.2.12.1 and BA.2.13, showed enhanced ACE2-binding affinity compared with BA.1. In contrast, BA.4 and BA.5 showed the weakest receptor binding activity owing to F486V and R493Q reverting mutations to the original wild type. However, compared with BA.2, BA.2.12.1, BA.4, and BA.5 showed enhanced immune escape in serum of 3-dose vaccinators (CoronaVac or ZF2001), especially in patients who recovered from BA.1 (Cao et al., 2022b).

A growing body of literature strongly supports that various single Omicron mutations could weaken neutralizing antibodies of different epitope groups, according to the escape mutation profiles (Cao et al., 2022a). The single mutations K417N, G446S, E484A, and Q493R largely bypass neutralizing antibodies whose epitopes overlap with the ACE2-binding motif (Cao et al., 2022a). Furthermore, single mutations of G339D, N440K, S371L, and S375F allow a subset of neutralizing antibodies to escape. Over 85% of studied human neutralizing antibodies are evaded, implying that Omicron may cause significant humoral immune evasion and antigenic shifting (Cao et al., 2022a). Evidence from cryo-electron microscope suggested that omicron S-trimers only had one structural state, with one "up" RBD and two "down" RBDs, whereas prior variations had uni-up and omni-down conformations.(Cui et al., 2022; Cerutti et al., 2022)

Some studies suggested that Omicron may have differentiated early from other SARS-CoV-2 variants (Planas et al., 2021a; Cameroni et al., 2021; Viana et al., 2022). It may originate from a period of gestation in immunocompromised individuals (such as patients with AIDS infected with the novel coronavirus) or evolve from non-human species and eventually flow back into humans (Kumar et al., 2021b). As of December 2021, related confirmed cases have been found in Australia, Belgium, Botswana, the United Kingdom, Denmark, Germany, Hong Kong, Israel, Italy, the Netherlands, and South Africa (del Rio et al., 2022). Later, SARS-CoV-2 mutant Omicron spreads and dominates worldwide (Nie et al., 2022). It is well known that neutralizing antibodies target the spike RBD (Yuan et al., 2021). There are multiple antigenic sites present in RBD, including RBS-A, RBS-B, RBS-C, and so forth. 15 mutations of Omicron can be located in the above antigenic sites (Zimerman et al., 2022). Whether Omicron can escape immune recognition is a matter of concern.

Moreover, a more recent study showed that the neutralizing epitope maps to the N2 and N3 loops in the N-terminal domain of the S protein (McCallum et al., 2021b). Another study confirmed that eight mutations in Omicron alter these epitopes and are associated with immune evasion (Javanmardi et al., 2021). Notably, HLA class II epitopes are disturbed by special mutations (Δ211/L212I, ins214EPE), resulting in damage to dendritic cell priming, which causes insensitive to the existing antibodies that interact with this region and immune evasion (Parker et al., 2021; Fang and Shi, 2022). One study also pointed out that there are other factors that affect the ability of a virus to spread besides receptor binding affinity (Fang and Shi, 2022). Therefore, mutations other than the S protein can also cause immune evasion of the novel coronavirus. More research is needed to explore the mechanism of other mutations on the immune evasion of novel coronavirus (Johnson et al., 2021; Fang and Shi, 2022). The spike of Omicron has displayed a decreased binding to dissolvable ACE2, and it may contribute to the lower efficiency of cell-cell fusion in Omicron strains (Zhao et al., 2022). Some studies have considered the fitness cost of RBD mutation accumulation under the selective pressure of nAb escape (Weisblum et al., 2020; Yi et al., 2021; Van Egeren et al., 2021). In addition, reducing cell-cell fusion inhibited cytotoxicity and may contribute to reduced virulence of Omicron variants (Zeng et al., 2021).

**Figure 4:**
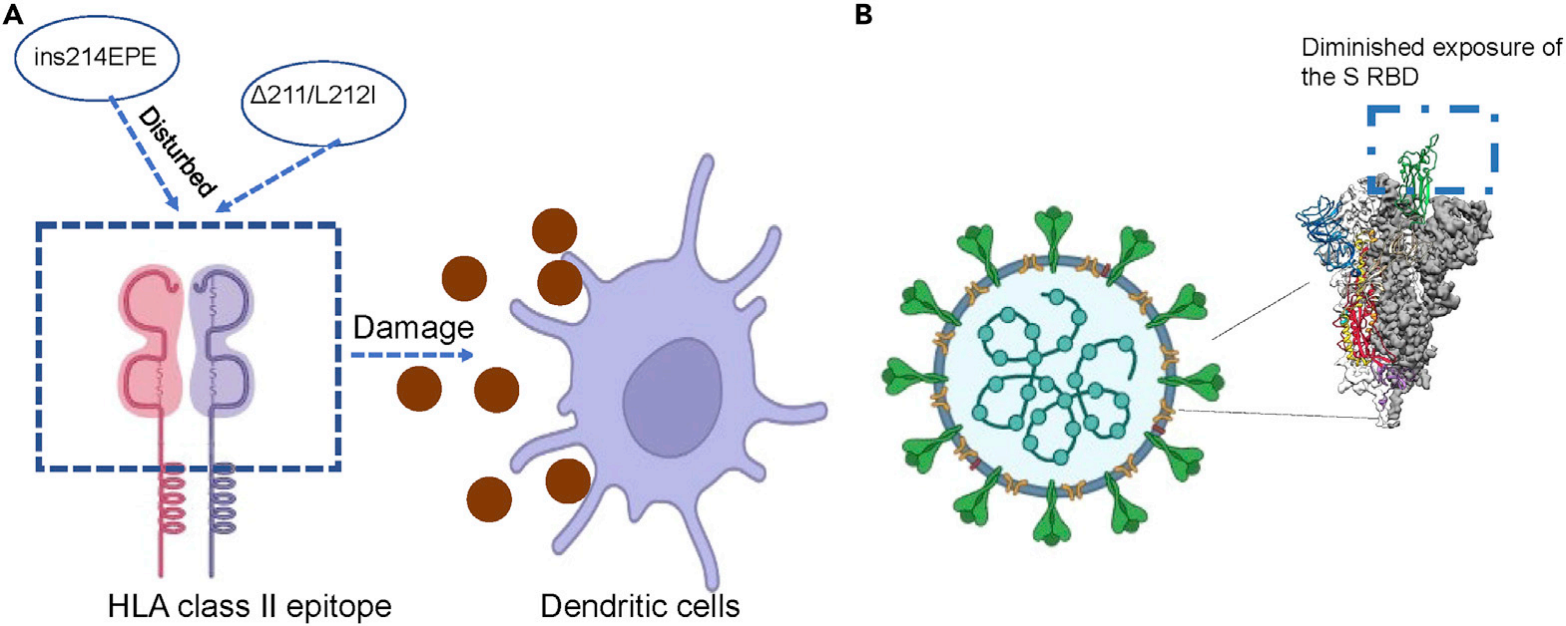
Immune escape mechanism of B.1.1.529 /Omicron (A) HLA class II epitopes are disturbed by special mutations (Δ211/L212I, ins214EPE), resulting in damage to dendritic cells and ultimately immune evasion; (B.1.1.529 /Omicron). (B) immune evasion by diminishing exposure of the S RBD.

Besides, accumulate evidence from clinical studies suggested that Omicron has more pronounced immune evasion compared with Delta variant, Gamma variant, and Alpha variant (Shuai et al., 2022). This study found that the Omicron spikes have some changes compared with previous variants, such as increased ACE2 binding and less dropping of the S1 subunit. But this strain has enhanced cell-to-cell transport, and homology modeling has confirmed a more stable closed S structure. The results indicated that Omicron has a bifold immune escape method owing to alterations of epitope and diminished exposure of the S RBD. In addition, omicron strains have enhanced spread owing to enhanced S protein consistency (Figure 4) (Shuai et al., 2022).

Investigations revealed that Omicron was unable to effectively utilize a protein on the cell membrane called "transmembrane serine protease 2" (TMPRSS2), which mediates the entry of viruses in certain cells. The authors suggested that mutations in the Omicron S protein may reduce its ability to utilize the TMPRSS2 pathway, thereby impairing its entry and replication in human epithelial cell lines (Zhao et al., 2022). Another study demonstrated that the researchers firstly expressed the Omicron spike Trimer in the pre-fusion state by *in vitro* recombination and analyzed the structure of spike Trimer under the conditions of normal physiological pH 7.5 and endosome pH 5.5 using cryo-EM (Cui et al., 2022). The structure showed that the spatial conformation of Omicron S protein is almost the same in the two pH environments, with one RBD in the "up" conformation and two RBDs in the "down" conformation.

In addition to the above-mentioned spatial state, the spike Trimer of WT and other VOC strains also have a state in which all three RBDs are in the "down" conformation. Besides, they found that Omicron spike Trimer has enhanced stability (Cui et al., 2022). Biochemical experiments also showed that the heat stability of Omicron S protein was higher than that of WT and Delta S protein. The increased stability of S protein improved the efficiency of recognizing receptor ACE2, but also reduced the subsequent fusion efficiency of the viral capsule and host cell membrane (Cui et al., 2022). Moreover, the neutralization activity of type 5 antibodies against Omicron was decreased. Through structural analysis, it was found that the deletion or mutation of amino acids in NTD and RBD regions of Omicron S protein led to great changes in antigen epitopes, resulting in the loss of neutralization activity of many neutralizing antibodies against Omicron, such as S477N on RBD (Cui et al., 2022).

Emerging variations have a number of key changes in the RBD that allow them to avoid immune monitoring and reduce the vaccine’s protective efficacy. The vaccine’s efficacy was slightly hampered by the Alpha variant’s single mutation (N501Y), and reinfection of the alpha was shown to be no worse than that of the prototype SARS-CoV-2 virus (Shen et al., 2021). Because it avoids part of the neutralising antibodies produced by the dominant strain, the beta variation poses a significant danger of reinfection. A critical residue related to increased immune escape rates is the E484K alteration on the -stained RBD. Despite the fact that only one amino acid (residue 417) varies in the RBD between the versions, it is more susceptible to spontaneously acquired or vaccine-induced antibody responses. Although the delta virus spread rapidly, there was little evidence that the vaccine was less effective. Omicron possesses the most mutations in the RBD’s receptor binding motif (RBM), where E484 and Q493 have been shown to play critical roles in immune escape. Immune escape occurred when E484A, Q493K, and Q493R recurred in immunocompromised patients or during monoclonal antibody treatment (Clark et al., 2021; Starr et al., 2021).

In comparison to the prototype SARS-CoV-2, inoculation with the dual mRNA1273 vaccine (non-boosted) and the supplementary BNT162b2-boosted vaccine lowered serum neutralization capacity of omicron by around 20-fold and 22.7-fold, respectively (Wilhelm et al., 2021; Han et al., 2022). In Omicron, the Q493R mutation is another key immune escape site that, when combined with local structural alterations produced by other surrounding mutations, leads to increased resistance to class I-III antibodies via steric conflict (Cui et al., 2022). Interaction between V445, G447, and P499 of RBD and Y35; V50, Y59, and Y105 HCDR from REGN10987, indicative of G446S substituted class IV antibodies; and mutant N440K with HCDR from HCDR3 disrupted the hydrophobic milieu. The antibody’s binding affinity to Omicron S is dramatically reduced by moderate conflict between Y102 (Cao et al., 2022a). According to one study, Omicron-specific RBD mutations increase their overall immunity to memory B-cell-derived antibodies (Sokal et al., 2022). Unsupervised clustering was used to split neutralizing antibodies into six epitope categories (A-F). The single alterations K417N, G446S, E484A, and Q493R generally avoided neutralizing antibodies in groups A-D (whose epitopes overlap with the ACE2 binding motif). Furthermore, single mutations of G339D, N440K, S371L, and S375F allowed a minority of neutralizing antibodies in groups E and F to escape (Cao et al., 2022a).

## Mutations of SARS-CoV-2 and vaccines

The Omicron variant is the latest variant of the novel coronavirus to emerge (WHO, 2021). The virus has been detected in more than 50 countries (Hoffmann et al., 2022). Because of the multiple mutations, scientists fear the Omicron variant strain could spread so quickly that the vaccine would not provide protection (Planas et al., 2021a). The study discovered that the fitness of Omicron BA.1 grew by 6.8 times and that of BA.2 increased by 8.88 times when compared to the earliest original wild type (Obermeyer et al., 2022). According to previous literature reports, a number of FDA-approved commercialized SARS-CoV-2 neutralizing antibodies almost lost their neutralizing activity against Omicron mutants. It is believed that Delta and Omicron variants spread quickly and carry a high viral load.

The NTD of the Delta and Omicron variants is greatly altered, which is responsible for its resistance to neutralizing antibodies (Viana et al., 2022). Its RBD also was changed, but this resulted in a small change in its antibody resistance. The Delta variant remained sensitive to several RBD-targeted antibodies. The variants all modify the NTD in different ways, changing its profile. RBD also was mutated, but the changes were more limited. The overall structure of RBD remains relatively stable in various variants, perhaps to preserve the ability of this S protein to bind to ACE2 receptors. Therefore, targeting RBD may be the most effective to focus the immune system on this key domain rather than the entire S protein (Thorne et al., 2022; Han et al., 2022). RBD is a more promising target for the next generation of vaccines and antibody therapies (Viana et al., 2022). The neutralizing antibodies (Abs) have been developed for targeting the NTD and RBD in infected patients. However, the S protein mutations reduced the neutralization potency of monoclonal Abs, Moderna mRNA-1273, Pfizer/BioNTech BNT162b2 (Bordon, 2021). Most of the S mutations were identified in the NTD and RBD regions. For example, Delta variants contain D614G, D950N, E156G, G142D, L452R, P681R, T19R, T478K substitutions as well as a residue deletion (157-158del) (Lopez Bernal et al., 2021). Kappa variants harbor D614G, E154K, E484Q, G142D, L452R, P681R, Q1071H, and T95I substitutions (Wang et al., 2021b). B.1.717.2+ (Delta plus) contains K417N mutation (Planas et al., 2021b). The appearance of COVID-19 vaccines has offered a huge hope for ending the pandemic. So far, most vaccine development focused on targeting the S glycoprotein of novel coronavirus (Rosenberg et al., 2021). Unfortunately, the efficacy of vaccines might be reduced because of the mutations of RBP and NTD domains in S protein. For instance, the Delta variant has raised concerns about the effectiveness of vaccines and the data revealed that the vaccine was less effective against the Delta variant compared to the previous variants (Lopez Bernal et al., 2021). Reports suggested that the Delta variant could easily mutate the N-segment structure and receptor domain of its S protein to escape antibody recognition (Augusto et al., 2021).

Moreover, some researchers analyzed the S protein sequences of β coronavirus that use ACE2 to recognize and infect host cells. Eleven conserved amino acids were found to be involved in binding ACE2, dividing them into four groups (Wang et al., 2021a; Cui et al., 2022). The four groups are: (1) group I consists of six very conserved amino acids G447, Y453, N487, Y489, T500, G502; (2) group II contains Y449/F/H, F456/L, Y473/F, F486/L, and Y505H; (3) group III includes G446/S/T, L455/S/Y, A475/P/S, G476/D, and G496/S; (4) group IV has five highly diverse residues, K417/V/N/R/T, E484/K/P/Q/V/A, Q493/N/E/R/Y, Q498/Y/H/R, and N501/Y/T/D/S (Cui et al., 2022). Some neutralizing antibodies, such as XGv47, A23-58.1, and S2K146, recognize group I-II amino acid epitopes, so these neutralizing antibodies can competitively bind S proteins with ACE2, and their antigen-binding epitopes are highly conserved (Wang et al., 2021a). It is expected to be a new generation of antibodies to treat and prevent Omicron and potentially novel coronavirus mutant strains.

According to one study, the omicron variation causes significantly less symptomatic disease than the delta variant. Vaccine effectiveness diminished quickly after two doses, with only minor vaccine effects visible 20 weeks following the second dose of any vaccine. Booster doses significantly increased protection against mild infection, but they also led to a decrease in protection against symptomatic sickness (Andrews et al., 2022). These findings demonstrate that Omicron breakthrough infections are less immunogenic than Delta infections, implying that they provide less protection against reinfection or infection by future variations (Servellita et al., 2022). Omicron has evolved a mutation that evades humoral immunity induced by BA.1 infection. The continued evolution of Omicron poses significant challenges to the establishment of an immune barrier for SARS-COV-2. This study suggests that BA.1-derived vaccine enhancers may not be ideal for achieving broad spectrum protection (Cao et al., 2022b). Emerging experimental and clinical data indicate that the efficacy of the main approved vaccine is significantly reduced against the Omicron variant, including BNT162b2 (Pfizer-BioNTech), mRNA-1273 (Moderna), Ad26.COV2.S (Johnson-Johnson), and ChAdOx1 nCoV-19 (Astra Zeneca). Serum from subjects fully inoculated with ChAdOx1 NCOV-19 or BNT162b2 showed little inhibition of Omicron variants (Ao et al., 2022).

Besides, a study published in Nature revealed that when plasma neutralization of Omicron was compared with that of the ancestral SARS-COV-2 strain, participants in both groups (Pfizer Vaccination vs Infection and Pfizer Vaccination) showed a 22-fold reduction in vaccine neutralization caused by the Omicron variant (Cele et al., 2022). Notably, a growing body of literature strongly suggested that in fully vaccinated individuals (mrNA-1273 vaccine), Omicron neutralization titers were reduced 49-84 times compared to D614G (Doria-Rose et al., 2021). However, most studies were carried out *in vitro*, more research on real viruses is needed to confirm the article’s findings in a more clinically relevant setting (Wang et al., 2021a).

Individuals were 89 percent, 87 percent, and 88 percent successful in preventing Alpha, beta, gamma, and Delta symptoms, respectively, seven days after receiving two doses of Pfizer. Individuals were 92 and 96% efficient in preventing Alpha and Delta symptoms, respectively, seven days following two doses of Moderna. Individuals were 91 and 87% successful in preventing Alpha and Delta symptoms, respectively, seven days following two doses of Astrazeneca (Nasreen et al., 2022). Individuals were 50%, 63%, and 57% successful in avoiding Beta, Gamma, and Delta symptoms 14 days after receiving a single dosage of Pfizer, respectively. Individuals were 82%, 89%, and 70% successful in preventing Alpha, Gamma, and Delta symptoms 14 days after taking one dose of Moderna, respectively. Individuals were 63%, 84%, 41%, and 68% successful in preventing Alpha, Beta, Gamma, and Delta symptoms 14 days after receiving a single dosage of Astrazeneca (Nasreen et al., 2022). Seven days after vaccination, two doses of Pfizer/moderna/astrazeneca were 82%, 80%, and 87% effective in avoiding hospitalizations and fatalities from the Alpha strain. Seven days after immunization, two doses of Pfizer/moderna/astrazeneca were 64%, 59%, and 61% effective in avoiding hospitalizations and fatalities from the Beta strain. Seven days after immunization, two doses of Pfizer/moderna/astrazeneca were 80%, 88%, and 89% effective in avoiding hospitalizations and fatalities from the Gamma strain. Seven days after immunization, two doses of Pfizer/moderna/astrazeneca were 81%, 90%, and 91% effective in avoiding hospitalizations and fatalities from the Delta strain (Nasreen et al., 2022).

Vaccine effectiveness was 65.5% (95% CI, 63.9 to 67.0) 2 to 4 weeks after the second dose, 15.4% (95% CI, 14.2 to 16.6) after 15 to 19 weeks, and 8.8% (95% CI, 7.0 to 10.5) after 25 weeks or more in individuals who had received two BNT162b2 doses. Vaccine effectiveness increased to 67.2% (95% CI, 66.5 to 67.8) among people who received BNT162b2 as the primary course from 2 to 4 weeks following a BNT162b2 booster dose before dropping to 45.7 percent (95% CI, 44.7 to 46.7) after 10 weeks or more (Andrews et al., 2022). During the omicron-predominant period, vaccine effectiveness was 40% (95% CI, 9 to 60) against hospitalization for Covid-19, 79% (95% CI, 51 to 91) against critical Covid-19, and 20% (95% CI, 25 to 49) against noncritical Covid-19 among adolescents 12 to 18 years old (median interval since vaccination, 162 days) (Price et al., 2022).

## Conclusion and future perspectives

**Figure 5:**
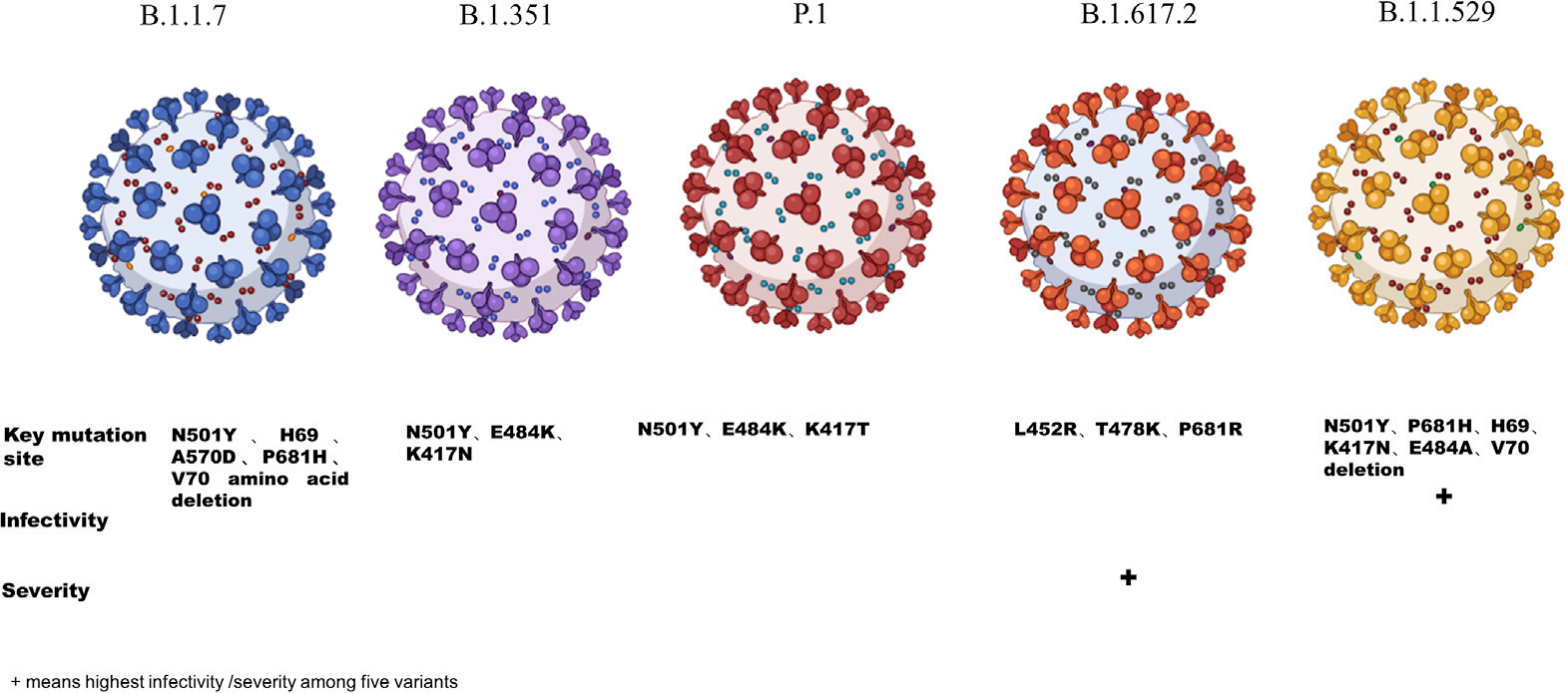
Key mutation sites, infectivity, and severity of the five variants

**Figure 6:**
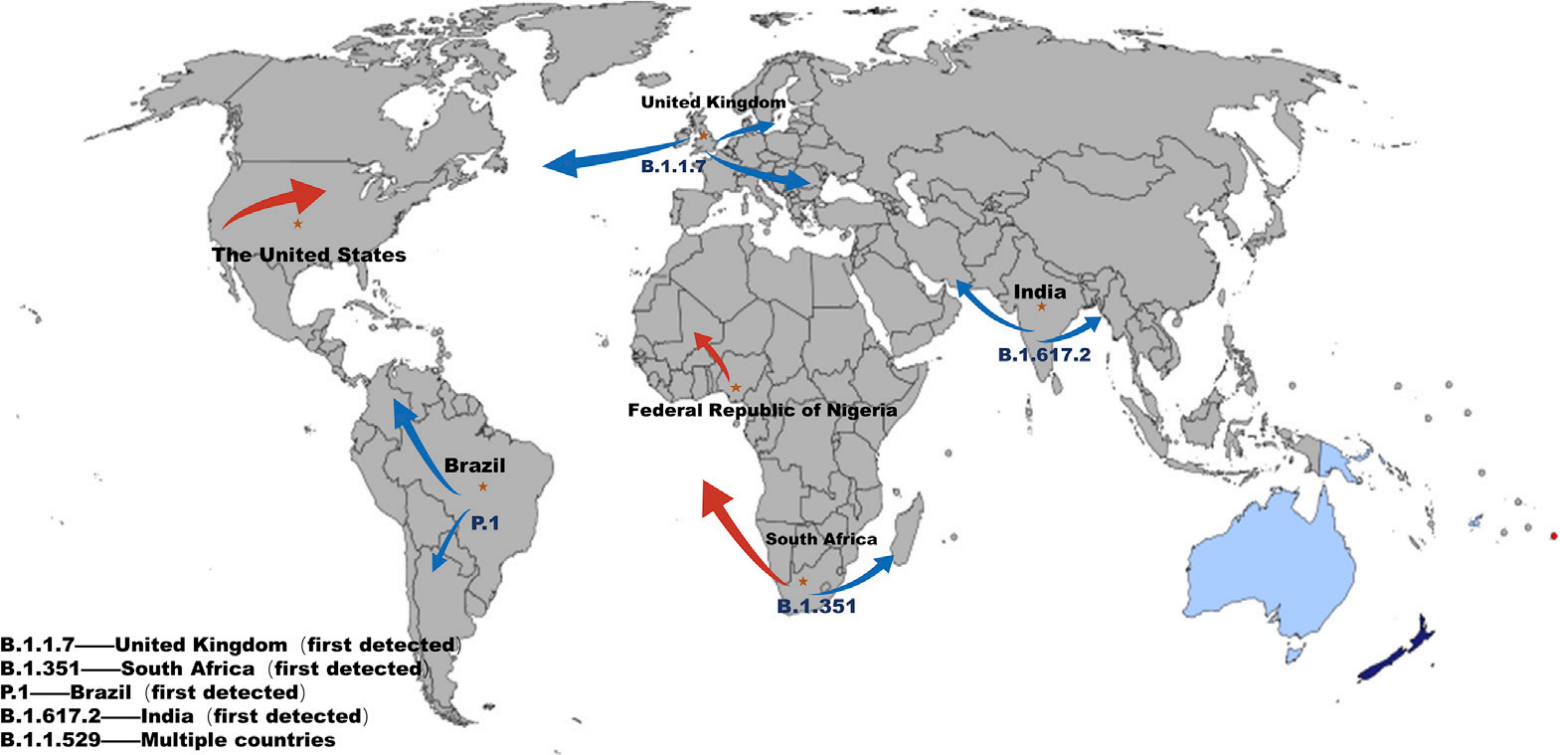
The original spread of different strains

So far, there are five variants of concern, including Alpha, Beta, Gamma, Delta, and Omicron. This article primarily discusses five variants: Omicron has the most mutations, with N501Y, P681H, H69, V70 deletion, K417N, E484A, and a total of 15 mutations on RBD. Delta has the most transmission power and the major mutation sites are L452R, T478K, and P681R. Although the death rate is unclear, the severe rate has risen. The N501Y, H69, V70 amino acid deletion, A570D, and P681H are the most common mutation sites. Beta will diminish the vaccine’s protection with the key mutation sites N501Y, E484K, and K417N. N501Y, E484K, and K417T are three significant Gamma mutation sites (Figures 5 and 6). The advent of a vaccine has raised hopes of ending the pandemic. However, unfortunately, the mutation variants have raised concerns about the effectiveness of vaccines and the data showed that the vaccine was less effective against mutation variants compared to the previous variants. In this article, immune escape mechanisms of several variants of concern are described. However, there are still many remaining issues that need to be resolved. It was demonstrated that CD4^+^ and CD8^+^ T cell responses are critical in the clearance of SARS-CoV-2 infection and COVID-19 infection, as well as modifying disease severity in people. Meanwhile, several SARS-CoV-2 mutations are likely to impact CD8-mediated responses (Rydyznski Moderbacher et al., 2020; Tan et al., 2021). One study indicated that the CD4^+^ T cell response is much lower in the Alpha, Beta, and Delta reference pools than in the overall pool. Spike protein explains the conserved CD4^+^ T cell response biologically, implying that the majority of CD4^+^ T cells target non-mutated areas in Alpha, Beta, and Delta variants (Mazzoni et al., 2022). It is necessary to investigate the impact of SARS-CoV-2 mutations on cellular responses. Probably the discovery of a wide range of neutralizing coronavirus antibodies will provide new clues for the development of new COVID-19 vaccines, and scientists will be able to design and develop a new generation of candidate vaccines, resulting in a wider range of immunity against coronavirus mutant strains. Moreover, vaccines targeting RBD may be more effective.

Meanwhile, further evidence suggested that convalescent plasma (CP) could be a new method for COVID-19 treatment. CP and hyperimmunoglobulin are taken from individuals who have recovered from infection and have developed an immune response to infected pathogens. Neutralizing antibody is considered the main active component. Other immune mediators may also play a role. Results from 10 severe adult cases showed that a single dose (200 mL) of CP was well tolerated and significantly increased or maintained high levels of neutralizing antibodies, leading to the disappearance of vireemia within 7 days. Meanwhile, the clinical symptoms and quasi-clinical criteria improved rapidly within 3 days. Imaging examination showed varying degrees of absorption of the lung lesions within 7 days (Duan et al., 2020). Another study also confirmed that convalescent plasma administration in COVID-19 reduced inflammatory markers (Klassen et al., 2021). Infusion of plasma with higher titer was associated with lower 30-day mortality compared with the infusion of plasma with lower antibody titer (22 vs. 30%) (Joyner et al., 2021). However, the convalescent plasma still needs to be tested on a large number of clinical samples. According to a case study, patients could develop anti-SARS-CoV-2 IgG 14 days after receiving convalescent plasma and patients with severe diseases were more likely to develop stronger antibody responses (Zhang et al., 2020). However, a randomized controlled trial of 333 patients with severe COVID-19 pneumonia showed that convalescent plasma therapy did not reduce 30-day mortality or improve other clinical outcomes compared with placebo (Simonovich et al., 2020).

Recently, several studies indicated that SARS-CoV-2 omicron is a special immune escape variant with a TMPRSS2-independent endosomal entry pathway (Willett et al., 2022). Moreover, SARS-CoV-2 spike mutations (N501Y and H655Y) are critical for the infection of aged and obese mice and escape from human immune sera (Rathnasinghe et al., 2022). Moreover, the lack of G446S mutation contributed to the sensitivity of BA.2 to BRD-5 antibody (Huang et al., 2022). Furthermore, evidence revealed that Omicron could evolve mutations to evade BA.1 infection-mediated humoral immunity, indicating that vaccine boosters from BA.1 variant could not protect the infection from new Omicron variants (Cao et al., 2022b). One study used vaccines and mAbs and naturally immune serum to explore the neutralization of BA.4/5 and found that BA.4/5 displayed reduced neutralization by the serum compared with BA.1 ad BA.2 (Tuekprakhon et al., 2022). Therefore, these escape mechanisms of BA.4/5 account for the possibility of repeat Omicron infection. In summary, in-depth investigations are necessary to determine the molecular mechanism of immune evasion of variants of concern and develop better vaccines for fighting COVID-19.

## Limitations of the study

Firstly, the included studies were time-limited. Variants of SARS-CoV-2 are rapidly developing, and some recent articles may not be cited timely. Secondly, many molecular mechanisms of SARS-CoV-2-induced immune escape have not been explored. To rapidly respond to pandemics caused by SARS-CoV-2 and future coronaviruses, it is necessary to continue studying their entry mechanisms, which could guide new therapeutic efforts. Lastly, further investigations are needed to design better vaccines based on immune escape mechanisms and viral properties, such as the conservation of fusion mechanisms and fusion subunit sequences, which make them potentially valuable targets for the development of pan-coronavirus therapeutics.

## Data Availability

The data presented in the study are included in the article. Further inquiries can be required from the corresponding authors.
